# High‐performance work systems, multiple commitments, and knowledge exchange and combination among Chinese public hospital nurses

**DOI:** 10.1002/nop2.921

**Published:** 2021-05-14

**Authors:** Bo Zhang, Fangcheng Tang, Bo Sun, Yi Niu, Zhisong Tang, Xiangdong Sun

**Affiliations:** ^1^ School of Economics and Management Beijing University of Chemical Technology Beijing China; ^2^ PLA Rocket Force Characteristic Medical Center Beijing China; ^3^ HRM Department Luoxin Pharmaceutical Group (Shanghai)Co., Ltd Beijing China

**Keywords:** Chinese public hospital nurse, high‐performance work systems, knowledge exchange and combination, multi‐foci commitment

## Abstract

**Aim:**

This research explores and examines the differentiated mediation roles of multi‐foci commitment in the relationship between HPWS and knowledge exchange and combination among Chinese hospital nurses.

**Design:**

This study employs a quantitative research approach and survey design.

**Methods:**

Individual‐level time‐lagged data were collected from 845 nurses in public hospitals in China using online questionnaires. Aside from personal information, the items in the questionnaires were adopted from mature scales in previous research.

**Results:**

The results of multiple regression analysis demonstrate that nurses’ professional and affective commitments partially mediate the relationship between HPWS and KEC. Nurses’ continuance commitment does not play a mediation role in HPWS‐KEC relationship, although HPWS positively influences nurse continuance commitment, which then weakly and positively impacts KEC.

## 1 INTRODUCTION

Nowadays, healthcare organizations strive to engage workers in knowledge exchange and combination (KEC) for innovative outcomes (Cousins et al., 2011; Rolls et al., 2020). Among healthcare professionals, nurse KEC is very important, because the knowledge of nurses is often the cornerstone of patient treatment and decision‐making (Hansen, Lauridsen, Camilla, & Løfgren, 2018). An effective way to engage healthcare workers in KEC is using modern human resource management (HRM) systems, or high‐performance work system(s) (HPWS)^1^ (Collins & Smith, 2006; Michaelis et al., 2015; Monks et al., 2016). HPWS is defined as “a system of HR practices designed to enhance employees’ skills, commitment, and productivity in such a way that employees become a source of sustainable competitive advantage” (Datta et al., 2005:136). This is especially the case in Chinese public hospitals (Zhou & Nunes, 2016). In the mid‐1990s, reforms reduced government financial support, decentralized the operational responsibilities to hospitals, increased the autonomy of managers and granted more latitude in HRM (Likun et al., 2000; Mei & Kirkpatrick, 2019; Pei et al., 2004). In 2011, the State Council issued guidelines on HRM issues concerned with remuneration and working conditions of healthcare staff. These and other directives have emphasized the need for public hospitals to improve efficiency, quality and cost control through improvements in their governance and internal management (Meng et al., 2015). Such a trend has been reinforced by “Guidelines on Establishing a Modern Hospital Management System” issued in July 2017 by the General Office of the State Council. Chinese public hospitals have been increasingly implementing HPWS and practices (Zhang et al., 2013). Therefore, in Chinese public hospitals, whether and how the use of modern HRM systems impacts nurses’ KEC have become topics that deserve scholarly attention, given that no such attention has been in evidence.

## 2 BACKGROUND

### 2.1 Literature review

HRM scholars have reported that HPWS influences employee KEC through mediators like social climate (Collins & Smith, 2006) and reflexivity (Monks et al., 2016). Yet little research has investigated whether a worker's commitment mediates the HPWS‐KEC relationship. This is surprising, because arguments and evidence in both HRM and knowledge management literatures have implied a mediation role of employee commitment in the HPWS‐KEC relationship. On the one hand, HPWS is conceptualized and reported to develop committed employees (e.g. Rode et al., 2016; Sanders & Yang, 2016). On the other, organizationally committed workers are likely to donate and collect knowledge at the workplace (e.g. Chiang et al., 2011; Li et al., 2017). The missing mediation role of worker commitment can seriously reduce the explanatory power of current theory about how HPWS influences KEC.

Moreover, workers may have a multi‐foci commitment to targets in the workplace (e.g. co‐workers, groups and the organization) (Perry et al., 2016; Swart et al., 2014; Wombacher & Felfe, 2017) and to other institutions or targets outside of work (e.g. family, professionals and social organizations) (Zhou et al., 2020). Some commitments are associated with the employee's higher‐order needs, while others are linked to lower‐order needs. However, HPWS can only shape the employee–organization relationship to offer employees higher‐order social resources (Liu et al., 2017). That said, HPWS may impact an employee's lower‐ and higher‐order commitments differently. Lower‐ and higher‐order commitments may impact an employee's KEC differently, because KEC is a behaviour associated with higher‐order rather than lower‐order needs (see also Liu et al., 2017 for an empirical example). HRM scholars (Cafferkey et al., 2017; Wright & Kehoe, 2008) have also been calling for achieving a better understanding of differentiated mediation roles of an employee's multi‐foci commitment in the HRM‐outcome relationship. Thus, this study's overall aim is to explore and examine the differentiated mediating effects of a nurse's multiple commitments in the HPWS‐KEC relationship. Specifically, it examines whether professional, affective and continuance commitment mediate the relationship between HPWS and KEC on the one hand. It examines how differently do the three commitments mediates the HPWS‐KEC relationship on the other hand.

### 2.2 An integrative theoretical framework

This study investigates the differentiated mediation roles of a nurse's professional, affective (organizational) commitment and continuance (organizational) commitments^2^ in the HPWS‐KEC relationship. Professional commitment refers to the extent to which professionals feel tied to their professional groups (Sollamia et al., 2018, p. 142). “Affective commitment refers to an emotional attachment to a firm such that the committed individual identifies with, is involved in, and enjoys membership in the firm, whereas continuance commitment refers to the tendency to stay in a firm on the basis of the potential loss or costs associated with leaving the firm” (Gong et al., 2009, p. 263).

This study further synthesizes the principles in social exchange theory (SET), the resource‐based view (RBV), the hierarchy of needs theory (Liu et al., 2017) and the multi‐foci commitment approach (Swart et al., 2014) to test the roles of multiple commitments in the HPWS‐KEC relationship. SET focuses on the resources that people obtain from, and contribute to, social interactions (Cropanzano & Mitchell, 2005). Exchange parties follow the principles of reciprocity and equivalence. Reciprocity means that the recipient is obligated to return a benefit to the party who provides such a benefit, while equivalence refers to the recipient returning benefits of equivalent value (Cropanzano et al., 2016). HRM systems can shape the nature of the employee–organization relationship (Liu et al., 2017; Tsui et al., 1997) to induce employee commitment (e.g. Heffernan & Dundon, 2016).

The hierarchy of needs theory differentiates an individual's needs into higher‐ and lower‐order categories. Based on the reciprocity and equivalence principles of SET, the social resources associated with an employee's higher‐order needs offered by the organization will induce an employee to reciprocate higher‐order attitudes and behaviours (Gong et al., 2009). Correspondingly, the social resources connected to an employee's lower‐order needs offered by the organization will induce an employee to reciprocate lower‐order attitudes and behaviours (Liu et al., 2017). An employee's professional and affective commitments are higher‐order commitments, because they both have affective and advancement components and increase motivation to produce, not just to stay. In comparison, continuance commitment is associated with lower‐order needs such as security (Gong et al., 2009). Therefore, when one type of HRM system shapes the employee–organization relationship to induce an employee's multiple commitments, an employee's experiences of the commitments may vary, because the type of HRM systems may offer social resources that can hardly be associated with both higher‐ and lower‐order needs.

From the RBV perspective, an organization's valuable and firm‐specific human resources contribute to sustainable competitive advantage (Barney & Wright, 1998; Jiang et al., 2013). Integrating this RBV logic into SET, the social resources offered by HRM system‐shaped employee–organization social exchanges are of a stronger organization focus than a profession focus. Based on the rationales of multi‐foci commitment, an employee's professional commitment is one of professional focus, while an employee's affective and continuance commitments are of an organizational focus. Therefore, HPWS may shape the employee–organization relationship to induce organization‐ and profession‐focused commitments differently. Further, compared with affective and professional commitment, employees with continuance commitment are barely valuable resources to an organization (Gong et al., 2009). As a result, HPWS may shape the employee‐organization relationship to induce affective and professional commitment rather than continuance commitment. In sum, the three commitments may impact KEC differently.

### 2.3 Hypothesis development

Although there is no consensus as to the elements of HPWS, they usually include HR practices such as comprehensive and developmental selection, developmental training programmes, performance appraisal, results‐ and skill‐based compensation, job rotation, career development, empowerment‐based job design and work teams (Lepak et al., 2006; Takeuchi et al., 2007; Xiao & Tsui, 2007).

The HPWS shapes the employee–organization relationship to offer nurses multiple social resources that can satisfy their higher‐order needs (Liu et al., 2017). Specifically, through participative decision‐making, nurses are offered opportunities for self‐expression and influence, are charged with greater responsibilities and have a greater sense of personal importance. Extensive selections grant nurses recognition and approval of the competence of those being selected (Blau, 1986), and training programmes offer nurses opportunities to achieve mastery of skills (Takeuchi et al., 2007). Such competence and skill mastery are also valuable in professional advancement. Career development practices extend an employer's care for and commitment to nurses’ futures in the organization and profession (Gong et al., 2009). Results‐based performance appraisal and job design based on individual skills and capabilities can grant nurses autonomy and job influence (Snape & Redman, 2010).

Further, when using participation practices, hospitals offer opportunities for nurses to hold shared objectives and to cooperate with co‐workers to achieve those objectives (Foss et al., 2015). Team‐based appraisal and profit‐sharing practices deliver shared responsibilities among co‐workers for work outcomes and their potential rewards. Working in such a situation, nurses are likely to develop trust with each other.

The multiplicity of systems of HR practices sets and reinforces the tone of the social exchange relationship with employees (Gong et al., 2009). Employees can perceive the self‐image, trust, empowerment, shared goals and responsibilities among co‐workers. In return, they are likely to reciprocate through generating higher‐order affective commitment (i.e. Gong et al., 2010 ). To reciprocate the opportunities to achieve skill mastery, improve self‐image and share goals and responsibilities among healthcare professionals, especially nurses, employees are likely to return their employer higher‐order professional commitment.

Continuance commitment is a lower‐order attitude, associated with lower‐order factors like security. HPWS, however, shapes employee–organization relationships to provide higher‐order social resources. Based on SET’s reciprocity and equivalence principles, HPWS can hardly induce nurses to experience continuance commitment. Therefore, we propose that:

**H1a:** *HPWS positively influences a nurse's* *professional commitment*.

**H1b:** *HPWS positively influences a nurse's* *affective commitment*.

When HPWS shapes the employee‐organization relationship to induce an employee's commitments, employees are likely to reciprocate hospital with different levels of affective and professional commitments. The rationale rests in the (mis‐)match of the focus of HPWS and the foci commitment (Swart et al., 2014). HPWS is one of strong organization focus. Hence, it aims to shape employee–organization relationships to offer social resources that primarily induce employees’ contribution to their organizations, rather than to other targets, such as professions (Jiang et al., 2013). An employee's professional commitment has a professional focus, while an employee's affective commitment has an organizational focus (Perry et al., 2016). Although these social resources are also useful in satisfying the needs of personal development in the profession, they are less effective in inducing an employee's professional development in comparison with self‐enhancement within the organization. Therefore, we propose that:

**H1c:** *HPWS has a stronger positive influence on a nurse's* *affective commitment than on professional commitment*.

### 2.4 The roles of a nurse's multiple commitments

Professionally committed employees have a strong aspiration to expand their skills or knowledge (London, 1983). They are also usually willing to make efforts, maintain membership and hold beliefs on goals and values (Lu et al., 2000, 2002). In particular, professionally committed employees aim to improve their knowledge and skills through exchanging and combining knowledge with colleagues in the same profession (Connelly et al., 2019; Connelly et al., 2011).

Affectively committed employees are identified with an organization and its members. Such a collective identity also develops a sense of shared purpose (Van Steenbergen & Ellemers, 2009), which encourages employees to exchange knowledge for the betterment of the organization (Priestley & Samaddar, 2007). Affectively committed employees also value organizational recognition and personal advancement. As a result, they are likely to make efforts in conducting KEC to improve personal image and improve knowledge and skills to achieve career progress within the organization.

Continuance commitment is associated with lower‐order motives and behavioural consequences (Whitener & Walz, 1993). It strongly influences knowledge combination and exchange, which is concerned with higher‐order pursuits, such as skill mastery and personal advancement (Lee et al., 2018). Similar findings have been reported in the literature. For example, Gong et al. (2009) found that continuance commitment of middle managers did not influence a firm's performance and did not mediate the relationship between maintenance‐based HRM and firm performance. Therefore, the research does not expect continuance commitment to impact an employee's KEC. Consequently, we also do not expect that continuance commitment mediates the HPWS‐KEC relationship.

**H2a:** *Professional commitment positively influences a nurse's KEC*.

**H2b:** *Affective commitment positively influences a nurse's KEC*.

A nurse's professional commitment may generate a stronger impact on KEC than does affective commitment. Professional commitment leads an employee to identify with people in the same professional community (Lee et al., 2000; Martin et al., 2000), while affective commitment ensures that he or she identifies with members in the work organization (Meyer et al., 2002). The scope of professional identification overlaps with the scope of commitment targets. In this study, we focus on nurses’ KEC among co‐workers within hospitals. To make progress in a profession, professionally committed employees are likely to conduct KEC with some of their co‐workers doing similar work in the same work organization (Morrow & Wirth, 1989; Perry et al., 2016), while affectively committed employees will conduct KEC with co‐workers more extensively if it is beneficial to the organization or to personal advancements within the organization (Meyer et al., 2002). Therefore, we propose that:

**H2c:** *Affective commitment influences a nurse's* *KEC stronger than does professional commitment*.

Based on the foregoing discussion, we further propose that HPWS impacts an employee's KEC through the employee's professional and affective commitments. Further, since HPWS‐shaped employee‐organization relationships offer social resources with a primary focus on the organization, we propose that the indirect influence of HPWS on KEC through affective commitment is stronger than that through professional commitment. We do not expect an employee's continuance commitment to play a mediation role in the HPWS‐KEC relationship. The reason is as discussed above, that continuance commitment is neither an antecedent of KEC nor a consequence of HPWS. Therefore, we propose that:

**H3a:** *A nurse's professional commitment mediates the relationship between HPWS and KEC*.

**H3b:** *A nurse's affective commitment mediates the relationship between HPWS and KEC*.

**H3c:** *The indirect relationship of HRM‐KEC through a nurse's affective commitment is stronger than that through a nurse's* *professional commitment*.

**FIGURE 1:**
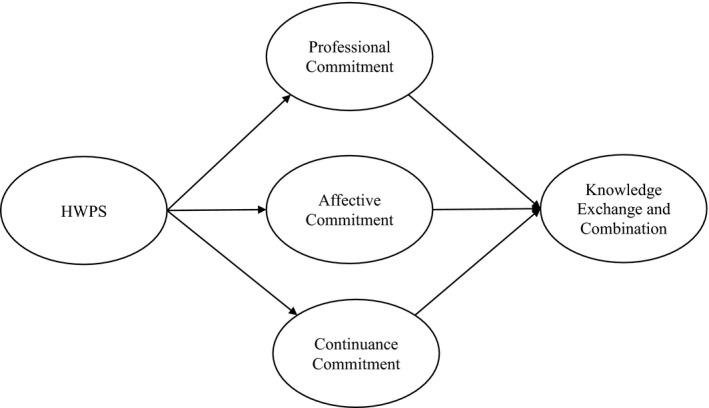
Conceptual model of the mediation role of a nurse's multiple commitments between HPWS and knowledge exchange and combination

Figure 1 provides a conceptual model of this study.

## 3 METHOD

### 3.1 Design and sample

This study employed a time‐lagged survey‐based approach. It collected individual‐level data about nurses in 20 public hospitals in Beijing city, Tianjin city and Hebei province. The selection of the 20 public hospitals follows a snowballing approach. At first, the corresponding author of this research contacted the HR head of 3 hospitals through personal relation network. Then, the HR heads of the 3 hospitals helped to contact other hospitals for access. All the researched hospitals are Grade‐A Tertiary Hospital, top‐tier hospital in China's hospital evaluation system (National Health Commission of the People’s Republic of China, 2011). All the nurses in the 20 hospitals were invited to participate in this research by completing the questionnaires. However, nurses had the right to decide whether to answer the questionnaires. Three online questionnaires were developed. The first questionnaire involves questions about the demographic details of the respondent and the questions about the experience of HPWS. The second questionnaire contains questions concerned with the three commitments of the respondent, while the third incorporates questions about the respondent's KEC. Each of the three questionnaires contains an ID code for matching the three waves of the data.

Since the scales used in the questionnaires were originally developed in English, a translation‐and‐back‐translation procedure was employed to guarantee the validity of the scale (Brislin, 1970). The translation‐and‐back‐translation of questions were conducted by the first author and the fourth author of this paper, who are both bilingual. Moreover, the first author has done translation‐and‐back‐translation of questionnaires in HRM and organizational behaviour field for many times. The fourth author has both academic experiences and extensive practical experience. It can guarantee the quality of the (back)translation and make sure the language used in questionnaires is colloquial. Before we conducted the survey, we invited 5 HR managers and 20 nurses to read and answer the questions. According to the feedbacks, all the questions can be well understood, and all the high‐performance work practices are used in their hospitals.

### 3.2 Data collection procedure

First, an invitation letter, coupled with the sample questionnaires, was sent to the HRM heads of the public hospitals to ask for consent for collecting the data. The researchers did not start collecting data until consent was granted by the HRM head or the top management team in the hospitals.

In May 2019 (Time 1), 1,600 nurses from 20 hospitals were invited to answer the first online questionnaire. A total of 1,127 nurses completed the questionnaire (a 70.44% response rate). Three weeks later (Time 2), the second online questionnaire was completed by 987 nurses (a 61.7% response rate). After another three weeks (Time 3), the third questionnaire was completed by 845 nurses (a 52.8% response rate). Hence, the final sample of this research contains 845 responses, with a response rate of 52.8%.

### 3.3 Measures

All items in the scales were evaluated on a 7‐point Likert scale (1 = strongly disagree, 7 =strongly agree). HPWS was measured using the 21‐item scale of Takeuchi et al. and’s (2007) research. Nurses were asked to evaluate their own experiences of high‐performance work practices. A sample item is “I received training continuously.” We formed an index of HPWS by taking the mathematic mean value of all the scores of 21 items/practices. Such way of treating HPWS as an index is widely accepted in the literature (Han et al., 2018; Jiang et al., 2012). Cronbach's alpha value was 0.833.

To measure the nurses’ multiple commitments, nurses were asked to evaluate “to what extent do you (dis)agree with the following descriptions?” Professional commitment was measured by using the 7‐item scale of Wang and Armstrong (2004), which was also used in other research (e.g. Perry et al., 2016). A sample item is “improve my knowledge in my field.” Cronbach's alpha value was 0.912. Affective commitment was measured by 7 items adopted from Meyer and Allen (1990). Cronbach's alpha value was 0.898. Continuance commitment was measured by 8 items adopted from Meyer and Allen (1990). Cronbach's alpha value was 0.924.

Knowledge exchange and combination of ideas were measured by using 8 items adapted from the scale of Collins and Smith (2006). The nurses were asked to evaluate “to what extent do you (dis)agree with the following descriptions?” A sample item is “at the end of each day, I feel that I have learned from others by exchanging and combining ideas.” Cronbach's alpha value was 0.897.

The study controlled the effects of variables, including a nurse's age, gender, tenure, education background and working sector in hospital, because these might impact the nurse's KEC (Monks et al., 2016).

### 3.4 Analysis

The data were analysed by using IBM SPSS version 21.0. We examined the means and standard deviations of the variables in the study, followed by Pearson's correlations to examine bivariate correlations among the variables.

Then, we examined the hypothesized relationships. In the first step, a series of hierarchical multiple regression analyses were conducted to examine the hypothesized relationships. Specifically, the three mediators (professional, affective and continuance commitment) were regressed on control variables (nurse's age, job tenure, education background, gender and hospital sector where the researched nurse work) and HPWS (see models 1, 2, 3 in Table 2 for details). In the second step, nurse's KEC was regressed on the control variables and HPWS (see models 4 and 5 in Table 2 for details). In the third step, nurse's KEC was regressed on the control variables and the three mediators (see model 6 in Table 2 for details). In the last step, nurse's KEC was regressed on the control variables, HPWS, and the three mediators (see model 7 in Table 2 for details) to examine the mediation relationships.

**TABLE 1:** Means, standard deviations and correlations between variables

	Mean	SD	1	2	3	4	5	6	7	8	9
Nurse's age	40.48	9.59									
Job tenure	19.89	11.29	0.828**								
Nurse's education background	–	–	0.099**	−0.143**							
Nurse's gender	–	–	0.081*	0.074*	0.161**						
Hospital Sector	–	–	−0.039	−0.010	0.021	0.042					
HPWS	5.23	0.80	0.150**	0.105**	0.037	0.062	0.029				
Knowledge exchange and combination	5.31	1.23	0.022	−0.050	0.098**	0.201**	0.017	0.321**			
Professional commitment	5.18	1.29	0.023	−0.020	0.009	0.149**	0.009	0.496**	0.334**		
Affective commitment1	5.58	1.11	−0.063	−0.110**	−0.046	0.057	0.038	0.306**	0.489**	0.308**	
Continuance commitment	4.98	1.34	0.017	0.021	−0.159**	0.020	0.022	0.642**	0.359**	0.456**	0.311**

Note

*N* = 845; SD = standard deviation.

* *p* < .05 (2 tailed).

** *p* < .01 (2 tailed).

## 4 ETHICS

Ethics approval was obtained from the research ethics committee of the business school where the research project was funded. Second, the top management team's or HRM head's official consent to access each of the hospitals was obtained before data collection. Third, each nurse could choose to participate or not in the research. Finally, participants were assured that the data would be used for academic research only and no individual information would be released.

**RESULTS**

Results of descriptive analysis included the mean value, standard deviation and correlation coefficient values of variables used in this paper (see Table1for details).

**TABLE 2:** The mediation effects of professional, affective and continuance commitment between HPWS and knowledge exchange and combination

	Professional commitment	Affective commitment	Continuance commitment	Knowledge exchange and combination			
	M1	M2	M3	M4	M5	M6	M7
	Standardized *β*	Standardized *β*	Standardized *β*	Standardized *β*	Standardized *β*	Standardized *β*	Standardized *β*
Control Variable							
Nurse's age	0.115	0.055	0.104	0.136*	0.003	0.025	−0.025
Job tenure	−0.189**	−0.135*	−0.250***	−0.123	−0.079	0.009	−0.026
Nurse's education background	0.018	−0.055	−0.115***	−0.198***	−0.198***	−0.172***	−0.191***
Nurse's gender	0.183***	0.133***	0.065*	0.049	0.017	−0.060*	−0.032
Hospital Sector	0.003	−0.009	0.031	0.028	0.006	0.019	0.006
Main Effect							
HPWS	0.312***	0.496***	0.316***		0.656***		0.532***
Mediators							
Professional commitment						0.224***	0.157***
Affective commitment						0.365***	0.141***
Continuance commitment						0.087**	0.016
*F* value	24.810***	51.456***	20.497***	5.688***	115.046***	44.936***	91.458***
*R* ^2^	0.145***	0.264***	0.122***	0.033***	0.452***	0.294***	0.491***
*△R* ^2^	0.095***	0.239***	0.097***	–	0.419***	0.268***	0.461***

Note

*N =* 845.

* *p *< .05

** *p* < .01

*** *p* < .001; VIF value ranges from 1.008–3.928.

All the variables used in regression analysis were centred to reduce non‐essential multicollinearity (Aiken et al., 1990). The results are exhibited in Table 2. The VIF value ranges from 1.008–3.928, indicating multicollinearity is not a concern in this study.

Hypothesis 1a states that HPWS positively influences a nurse's professional commitment. Hypothesis 1b states that HPWS positively influences a nurse's affective commitment. Hypothesis 1c states that HPWS has a stronger positive influence on an employee's affective commitment than does professional commitment. In Table 2, the results in models 1, 2 and 3 show that nurse‐experienced HPWS was positively and significantly related to professional (*β* = 0.312, *p* < .001) and affective (*β* = 0.496, *p* < .001) commitments. Therefore, Hypotheses 1a and 1b were supported. It is also demonstrated that HPWS impacts affective commitment most strongly. Hence, Hypothesis 1c was also supported. Surprisingly, HPWS was found to positively impact a nurse's continuance commitment (Table 2, Model 3, *β* = 0.316, *p* <. 001).

H2a states that a nurse's professional commitment positively influences KEC. H2b states that a nurse's affective commitment positively influences KEC. H3c states that a nurse's affective commitment influences KEC stronger than does professional commitment. In Table 2, the result in Model 6 shows that a nurse's KEC is positively and significantly influenced by the nurse's professional (*β* = 0.224, *p* < .001) and affective (*β* = 0.365, *p* < .001) commitment. Therefore, both Hypotheses 3a and 3b were supported. However, contrary to our expectation, continuance commitment positively and significantly impacted a nurse's KEC, although the correlation coefficient was very small (Table 2, Model 6, *β* = 0.087, *p* < .001).

H3a states that a nurse's professional commitment mediates the relationship between HPWS and KEC. H3b states that a nurse's affective commitment mediates the relationship between HPWS and KEC. In Table 2, the results in Model 5 show that HPWS significantly and positively impacts a nurse's KEC (*β* = 0.656, *p* < .001). When HPWS and the three types of commitments were entered in the analysis together, it was found that the impact of HPWS on a nurse's KEC became weaker but remained statistically significant (*β* = 0.532, *p* < .001). Both professional commitment (*β* = 0.157, *p* < .001) and affective commitment (*β* = 0.141, *p* < .001) remain significantly and positively related to a nurse's knowledge exchange and combination of ideas, while a nurse's continuance commitment was no longer significantly related to the nurse's KEC. Therefore, a nurse's professional and affective commitment partially mediated the relationship between HPWS and KEC. As a result, both Hypotheses 3a and 3b were partially supported. As expected, continuance commitment did not mediate the HPWS‐KEC relationship. Hypothesis 3c states that the indirect relationship of HPWS‐KEC through a nurse's affective commitment is stronger than that through a nurse's professional commitment. The effect size of the indirect relationship of HPWS‐KEC through professional commitment is calculated by multiplying the effect size of HPWS on professional commitment by the effect size of professional commitment on KEC (0.312 × 0.157 = 0.049). In the same way, we calculate the effect size of the indirect relationship of HPWS‐affective commitment‐KEC, which is 0.496 × 0.141 = 0.070. Therefore, Hypothesis 3c was also supported.

## 5 DISCUSSION

The overall purpose of this paper is to examine the differentiated mediating effects of nurses’ professional, affective and continuance commitment in the relationship between HPWS and nurse's KEC. The findings generate both theoretical and practical implications.

### 5.1 Theoretical implications

First, the usefulness of HPWS in engaging nurses in KEC has been reported in studies based on other sectors, such as knowledge‐ and skill‐based workers in high‐technology firms, in developed countries such as the United States (e.g. Collins & Smith, 2006), Ireland and the UK (e.g. Monks et al., 2016). This research adds new evidence about HPWS’s effectiveness in encouraging nurses to conduct KEC in the Chinese healthcare sector.

Second, HPWS was positively related to both professional and affective commitments among nurses in Chinese public hospitals. These findings are in line with the conceptualizations and empirical evidence in the HRM literature that HPWS or performance‐oriented HRM systems positively influence employee outcomes associated with higher‐order needs (e.g. Gong et al., 2009; Liu et al., 2017). This research further found that HPWS exerted a stronger impact on affective commitment than it did on professional commitment. The finding supports the theoretical logic of strategic HRM that HPWS offers social resources that induce an employee's organization‐specific contributions to gain competitive advantage. Differing from previous research, which examined how different HRM systems impact different organization‐focused commitments differently (e.g. Gong et al., 2009), this research is the first to investigate how one HRM system, that is HPWS, differently impacts two higher‐order commitments differently. It contributes to the literature by revealing that, besides different needs (higher‐ or lower‐order) associated with employee attitudes and behaviours, the multiple foci (organization vs. profession) of pursuits also vary the effectiveness of HPWS.

Third, contrary to our expectation, HPWS was found to be positively related to a nurse's continuance commitment. However, this situation might be plausible. According to Meyer et al. (1993), “continuance commitment presumably develops as employees recognize that they have accumulated investments or ‘side bets’ (H. S. Becker, 1960) that would be lost if they were to leave the organization” (p. 539). The HPWS‐shaped employee–organization relationship offers nurses social resources associated with their higher‐order needs or pursuits. Nurses as “the recipients of social resources might … believe that to lose such a package would be costly, and therefore experience greater continuance commitment” (Meyer & Smith, 2000, p. 320). Moreover, in the HPWS‐shaped employee–organization relationship, nurses and organization mutually invest in each other. Leaving the organization means that nurses will lose the potential return of their previous investments and efforts.

Fourth, both professional commitment and affective commitment were positively related to a nurse's KEC. The findings are in line with RBV principles that professionally and affectively committed employees are valuable resources in creating sustainable competitive advantage (Barney, 1991; Gong et al., 2009). Moreover, compared with professionally committed nurses, affectively committed nurses conducted more KEC at work. This finding further reinforces that HPWS focuses mainly on offering social resources that can induce organization‐focused contribution. It was also found that a nurse's continuance commitment was positively and weakly related to KEC. This finding is out of our expectation and different from the argument (March & Simon, 1958) and finding of previous studies (Gong et al., 2009; Meyer et al., 2002; Shore et al., 1995). However, it may be rational for nurses with high continuance commitment to generate high performance. The motivation of these nurses is to stay and avoid loss or cost. If the condition of staying is generating a certain type and level of performance, these nurses will have to produce that performance above the required level (Meyer et al., 1990). In the situation of this study, the work in hospital necessitated knowledge exchanges through discussions with doctors or other healthcare staff.

Fifth, although one nurse can have multiple commitments stimulated by HPWS, not all these commitments can facilitate the influences of HPWS on KEC. It was found that a nurse's higher‐order professional and affective commitment partially mediated the influences of HPWS on the nurse's knowledge combination and exchange. However, the lower‐order continuance commitment did not mediate the HPWS‐nurse knowledge combination and exchange. Such findings are similar to previous findings (Gong et al., 2009). For example, Gong et al. (2009) found that a maintenance‐oriented HR system induced a lower‐order continuance commitment that did not impact performance. Differently and interestingly, although this study found both a positive HRM‐continuance commitment relationship and a positive continuance commitment‐KEC relationship, the indirect HPWS‐KEC relationship through continuance commitment was not significant. This seems strange at first glance. However, the empirical evidence does support the strategic HRM logic. HPWS, in shaping the employee‐organization relationship, offers social resources that can satisfy a nurse's higher‐order needs and consequently induce the nurse's higher‐order commitments. Nurses with higher‐order commitment are valuable resources for an organization to produce sustainable competitive advantage. Although HPWS did increase a nurse's continuance commitment, this is likely to be the by‐product of creating an organization‐specific employee–organization relationship. In other words, the purpose of nurses with high continuance commitment in conducting KEC is to meet the organization's requirements for staying rather than proactively to manage knowledge. Thus, HPWS can strongly impact a nurse's KEC through cultivating the nurse's continuance commitment.

Lastly, the indirect HPWS‐KEC relationship through affective commitment was stronger than that through professional commitment. This again is aligned with strategic HRM theory that HPWS cultivates organization‐specific human resources, which contributes primarily to organization‐focused outcomes.

### 5.2 Practical implications

This research can also generate several practical implications for decision‐makers in hospitals. On the one hand, a series of HRM practices, such as employee empowerment, extensive training, team‐based performance appraisal and pay, etc., could be used together to form a HPWS to encourage public hospital nurses to exchange and combine knowledge.

On the other hand, since professional and affective commitment mediates the HPWS‐KEC relationship significantly, additional managerial tools should be adopted to further complement HPWS, for example building internal and external network building. Although continuance commitment positively impacts nurse's KEC, the impact is marginally and could be attributed to the bottom‐line requirements of nurse's daily work. Given previous studies have reported the detrimental effects of continuance commitments such as attendance, task performance and helping behaviours (Meyer et al., 2002), it is advisable to use additional managerial tools to reduce continuance commitment's potential negative effects and strengthen its positive effects. For example, specifically protocol about knowledge exchange and combination activity and evaluation procedures could be employed.

### 5.3 Limitations and suggestions for future research

The study acknowledges that there are limitations. First, although the study collected data at three different time points, it employed a single‐respondent approach. Future research is urged to use multiple sources when collecting data. Second, this study tested the mediation role of a nurse's multiple commitments between HPWS and the nurse's KEC. This is a good starting point. Future research is encouraged to explore the boundary conditions of such indirect or direct effects of HPWS on KEC. Future research is also suggested to investigate the interactive effects between the different types of commitment in determining knowledge management outcomes. Finally, although it was found that all the three types of commitments were positively related to a nurse's KEC, they are likely to impact a broader scope of an employer's expected or unexpected work outcomes. Future studies could further investigate the effects of multiple commitments on other outcomes.

## 6 CONCLUSION

The conceptualization and empirical results may enhance our understanding about the differentiated roles played by a nurse's multiple commitments in the HPWS‐KEC relationship. On the one hand, our results demonstrate the differentiated effects of HPWS on the three types of commitments, and the differentiated impacts of the three commitments on KEC. A nurse's higher‐order professional and affective commitments mediate the relationship between HPWS and KEC, while the lower‐order continuance commitment does not. Although HPWS positively impacts continuance commitment, which positively and weakly impact KEC, continuance commitment does not mediation the HPWS‐KEC relationship. One the other hand, between the two higher‐order commitments, affective commitment plays a stronger mediation role than does professional commitment in the HPWS‐KEC relationship.

## CONFLICT OF INTEREST

No conflict of interest has been declared by the authors.

## AUTHORS CONTRIBUTIONS

The first author conceived and designed the study. The first, third and sixth authors collected the data. The sixth author conducted data analysis. All the authors contributed to manuscript writing. All authors have agreed on the final version and meet at least one of the following criteria (recommended by the ICMJE [https://www.icmje.org/recommendations/]): substantial contributions to conception and design, acquisition of data or analysis and interpretation of data; drafting the article or revising it critically for important intellectual content.

## Data Availability

Due to the nature of this research, participants of this study did not agree for their data to be shared publicly, so supporting data are not available. Please contact the corresponding author for any inquiries.

## ENDNOTES

^1^
In the literature, the terms ‘high commitment’, ‘high involvement’ and ‘high performance’ are often used interchangeably (Leggat et al., 2010).

^2^
Normative commitment is excluded from this study, based on social exchange theory, because “... [normatively] committed individuals may exhibit certain behaviors not because they have figured that doing so is to their personal benefit, but because they believe that it is the ‘right’ and moral thing to do” (Wiener, 1982, p. 421).
